# Match and Training Load Exposure and Time-Loss Incidence in Elite Rugby Union Players

**DOI:** 10.3389/fphys.2019.01413

**Published:** 2019-11-19

**Authors:** Ben E. W. Cousins, John G. Morris, Caroline Sunderland, Anthony M. Bennett, Golnaz Shahtahmassebi, Simon B. Cooper

**Affiliations:** ^1^Sport Performance Research Group, Sport Science Department, Nottingham Trent University, Nottingham, United Kingdom; ^2^Applied Sports, Technology, Exercise and Medicine Research Centre (A-STEM), College of Engineering, Swansea University, Swansea, United Kingdom; ^3^Department of Physics and Mathematics, Nottingham Trent University, Nottingham, United Kingdom

**Keywords:** RPE, GPS, exponentially weighted moving average, acute:chronic workload ratio, monitoring, mixed effect models

## Abstract

**Objective:**

To investigate the impact of match and training load on time-loss incidence in elite, professional Rugby Union players.

**Materials and Methods:**

Eighty-nine Rugby Union players were monitored over two seasons of training and competition. Load was measured for all training sessions and matches using subjective [session ratings of perceived exertion (sRPE) load; RPE × session duration] and objective [global positioning systems (GPS); distance and high-speed running distance] methods and quantified using multiple approaches; absolute match and training load, acute:chronic workload ratio (ACWR), exponentially weighted moving average (EWMA) and cumulative 7, 14, 21, and 28 d sums. Mixed effect models were used to assess the effect of each variable on time-loss incidence.

**Results:**

Of the 474 time-loss incidences that occurred across the two seasons, 50.0% were contact injuries (86.5% occurred during matches and 13.5% during training), 34.8% were non-contact injuries (31.5% occurred during matches and 68.5% during training) and 15.2% were cases of illness. The absolute match and training load variables provided the best explanation of the variance in time-loss incidence occurrence [sRPE load: *p* < 0.001, Akaike information criterion (AIC) = 2936; distance: *p* < 0.001, AIC = 3004; high-speed running distance: *p* < 0.001, AIC = 3025]. The EWMA approach (EWMA sRPE load: *p* < 0.001, AIC = 2980; EWMA distance: *p* < 0.001, AIC = 2980; EWMA high-speed running distance: *p* = 0.002, AIC = 2987) also explained more of the variance in time-loss incidence occurrence than the ACWR approach (ACWR sRPE load: *p* = 0.091, AIC = 2993; ACWR distance: *p* = 0.008, AIC = 2990; ACWR high-speed running distance: *p* = 0.153, AIC = 2994).

**Conclusion:**

Overall, the absolute sRPE load variable best explained the variance in time-loss incidence, followed by absolute distance and absolute high-speed running distance. Whilst the model fit using the EWMA approach was not as good as the absolute load variables, it was better than when the same variables were calculated using the ACWR method. Overall, these findings suggest that the absolute match and training load variables provide the best predictors of time-loss incidence rates, with sRPE load likely to be the optimal variant of those examined here.

## Introduction

It has been demonstrated in a number of professional sports, including Soccer (Carling et al., 2015) and Rugby Union (Williams et al., 2015), that success is inversely related to injury incidence, suggesting that player availability is a key determinant of success. Rugby Union has one of the highest reported incidences of match injury amongst all professional team sports, at 81 injuries per 1000 player hours for matches and 3 injuries per 1000 player hours for training (Williams et al., 2013). It is therefore crucial that Rugby Union coaches, performance and medical staff develop strategies to reduce time-loss incidence and maximize squad availability, thus enhancing the chances of team success.

Time-loss incidences are typically classified as either injuries or illness, with injuries further categorized as contact and non-contact (Fuller et al., 2008). In elite sport, the careful management of match and training load to minimize time-loss incidence, is a key role of performance, medical and coaching staff (Gabbett and Ullah, 2012; Rogalski et al., 2013; Blanch and Gabbett, 2015; Cross et al., 2016). Improper load management can negatively affect numerous physiological systems including the neuroendocrine, immunological, cardiovascular and musculoskeletal systems (Adams and Kirkby, 2001), resulting in an increased occurrence of time-loss incidence.

Research exploring the effects of match and training load on time-loss incidence rates has typically quantified load using either subjective or objective measures. Subjective measures of match and training load include ratings of perceived exertion (RPE), with the most commonly used outcome variable being session ratings of perceived exertion load (sRPE), calculated by multiplying session RPE (Borg CR10 scale; Foster et al., 2001) by session duration (in min) (Gabbett, 2004; Gabbett and Domrow, 2007). Objective measures include micro technology such as global positioning systems (GPS), which provide information such as the overall distance covered by the players in a given training session or match and the speeds at which those distances are covered (Colby et al., 2014). In recent years, research has been undertaken investigating the relationship between match and training load and time-loss incidence across a variety of sports, including Australian Rules Football (Rogalski et al., 2013), Rugby League (Blanch and Gabbett, 2015; Hulin et al., 2016), Cricket (Hulin et al., 2016), and Soccer (Bowen et al., 2016). One of the first papers to examine the relationship between match and training load and injury was conducted in 46 elite Australian Rules footballers (Rogalski et al., 2013). sRPE load showed that high training loads over 1 week of >1750 arbitrary units (AU) (compared to a reference group of <1250 AU) resulted in an increased occurrence of injury [odds ratio (OR) = 2.44–3.38]. Two week loads of >4000 AU (compared to <2000 AU) were also associated with an increased occurrence of injury (OR = 4.74), as were large changes (from 1 week to the next) of greater than 1250 AU (compared to a change of <250 AU; OR = 2.58). A more objective approach has also been used in Australian Rules Football, where GPS derived running loads and injury occurrence were assessed across one season of competition (Colby et al., 2014). Total distance and sprint distance were analyzed as cumulative 3-week loads and results showed that total distance between 73,721 and 86,662 m (compared to <73,721 m) increased the occurrence of non-contact injury (OR = 5.49), as did a high sprint (greater than 75% of the individual’s maximum velocity) distance (>1453 m compared to <864 m; OR = 3.67) (Colby et al., 2014). However, this objective approach to match and training load quantification has only been examined in Australian Rules football.

A number of different methods of quantifying match and training load have also been reported in the literature to date, including the acute:chronic workload ratio (ACWR) (Blanch and Gabbett, 2015) and the exponentially weighted moving average (EWMA) (Williams et al., 2017). The ACWR is the ratio of average load in the past 7 d (acute) compared to the average of the past 28 d (chronic) (Blanch and Gabbett, 2015); which when applied to sRPE load data, it has led to the suggestion of a “sweet-spot” (i.e., the match and training load associated with the lowest time-loss incidence risk) of 0.8–1.3 (80–130% in the past 7 d compared to the past 28 d). It is also interesting to note that the risk of injury increases when the ACWR goes above 1.5 (Blanch and Gabbett, 2015). In more recent years researchers have questioned the rolling average approach of the ACWR (Drew and Purdam, 2016; Lolli et al., 2017, 2018; Menaspà, 2017; Williams et al., 2017), with the suggested new approach to place greater weighting on the load completed in the acute phase (compared to the preceding days/weeks), due to the decaying nature of fitness and fatigue effects over time (Williams et al., 2017). This approach, defined as the EWMA, mitigates the issues described by Menaspà (2017) and Lolli et al. (2017), such as mathematical coupling, and is therefore potentially suggested as a more sensitive measure.

One of the few studies to explore the influence of in-season training loads on injury risk specifically in professional Rugby Union was undertaken by Cross et al. (2016). sRPE load was examined across four teams (*n* = 173 players) for the in-season period of one season of competition. Results showed that injury risk increased when 1-week load was 1245 AU greater than an average week (OR = 1.68) and when week-to-week changes in load exceeded 1069 AU (compared to no change, OR = 1.58). Furthermore, a likely harmful effect was seen when 4-week cumulative loads >8651 AU (compared to <3684 AU; OR = 1.39). However, the study by Cross et al. (2016), did not account for the loads accumulated from matches, which is typically the player’s biggest load in a week. Additionally, no objective measures were used to quantify load, therefore, no external load measurement was obtained, and training load was assessed in its absolute form, with no ACWR or EWMA quantification applied.

Therefore, the aims of this study were to examine and identify relationships between match and training load, derived through both subjective and objective measures, and time-loss incidence rates in elite Rugby Union players, across two seasons of competition. The study sought to identify the best predictor of time-loss incidence occurrence between absolute match and training load variables, the ACWR and the EWMA quantification methods. Furthermore, it was hypothesized that due to the decaying nature of fitness and fatigue, the EWMA approach to match and training load quantification would better explain the variance in time-loss incidence occurrence in comparison to the ACWR method. It was also hypothesized that the acute (last 7 d) period of match and training load would be the greatest predictor of time-loss incidence occurrence compared to the longer 14, 21, and 28 d timeframes.

## Materials and Methods

### Study Design

The study was a two-season prospective cohort study of Rugby Union players (*n* = 89, age: 26.5 ± 4.5 years, body mass: 104.3 ± 13.5 kg, height: 1.86 ± 0.07 m) registered in the first team squad of an elite professional English Rugby Union club, playing in the top two tiers during the 2016–2017 and 2017–2018 seasons. Ethical approval was provided by the host institution’s Ethical Advisory Committee and all players provided their written consent to participate. In brief, the quantification of load was undertaken using three methods; the absolute match and training load (cumulative daily load), the ACWR (Blanch and Gabbett, 2015) and EWMA match and training load ratio (Williams et al., 2017), with these calculations applied to subjective (sRPE load) and objective (GPS) data. Additional match and training load quantification was undertaken in the format of cumulative rolling sums for 7, 14, 21, and 28 d periods, again for both sRPE load and GPS data.

### Rating of Perceived Exertion

For every field- and gym-based training session and match, an RPE rating, using the modified Borg CR-10 RPE scale (Foster et al., 2001), was obtained within 30 min of the end of the exercise, in line with the recommendations of Kraft et al. (2014). Session RPE load in AU for each player was then calculated by multiplying the given RPE by the session duration (min) (Foster et al., 2001). This was performed for all players across both seasons of data collection. Session RPE load has previously been shown to be a valid method for estimating relative exercise intensity (Impellizzeri et al., 2004). The ACWR and EWMA calculations were then applied to the RPE data, yielding two variables: ACWR sRPE load and EWMA sRPE load. In addition, cumulative 7, 14, 21, and 28 d sums were calculated.

### Global Positioning Systems

An objective measure of match and training load was obtained through GPS for every field-based training session and match, for 33 out of the 60 players in the squad for season one and for all 56 players in season two. Overall, 27 players completed both seasons and 62 players completed only one of the two seasons. Two GPS systems were used (Catapult OptimEye S5 monitoring system, 10 Hz, Canberra, Australia, *n* = 18; and GPSports SPI-Pro, 5 Hz, Canberra, Australia, *n* = 15) during season one, with each player using the same GPS unit for the entire season. In season two all 56 players used the same GPS system (STATSports APEX, 10 Hz, Newry, Northern Ireland, *n* = 56). The number of satellites was satisfactory on all days for all systems, with an average of 9 ± 1 satellites per day being used and a horizontal dilution of precision of 0.58 ± 0.06. The firmware of the systems was the same for all units for the respective manufacturer and the firmware was not updated at any stage during the study. The manufacturer’s software was used to download all sessions and again the software was not updated at any stage during the study. Previous research has demonstrated the reliability and validity of each of the GPS systems used (GPSports SPI-Pro: Waldron et al., 2011; Catapult OptimEye S5: Thornton et al., 2019; STATSports APEX: Beato et al., 2018). Furthermore, the analysis of the distance covered at high speed has been shown to be associated with increased risk of lower body soft-tissue injury (Gabbett and Ullah, 2012). High-speed running was determined as the distance covered at greater than 70% of a player’s maximum velocity, determined during pre-season testing (40 m sprint testing) and updated if bettered at any stage across the season. GPS data were also quantified using ACWR and EWMA, giving rise to four further variables (ACWR distance, EWMA distance, ACWR high-speed running distance and EWMA high-speed running distance) and the cumulative 7, 14, 21, and 28 d rolling sums calculated for both distance and high-speed running distance.

### Data Handling

The ACWR was calculated as the average load of the previous 7 d divided by the average load of the previous 28 d (Blanch and Gabbett, 2015), with the acute, 7 d period also included in the chronic, 28 d period. The EWMA for any given day is calculated by; EWMA_today_ = Load_today_ × λ_a_ + ((1 − λ_a_) × EWMA_yesterday_) where λ_a_ is a value between 0 and 1 that represents the degree of decay. The λ_a_ is calculated; λ_a_ = 2/(*N* + 1), where *N* is 7 d (acute) or 28 d (chronic) time period, with the acute EWMA then being divided by the chronic EWMA to provide a single EWMA value. The absolute match and training load variables for sRPE load, distance and high-speed running distance, along with the aforementioned ACWR and EWMA variables were used in the analysis. Cumulative 7, 14, 21, and 28 d rolling sums for sRPE load, distance and high-speed running distance were calculated for each player. Due to the ACWR and EWMA variables requiring at least 28 d of match and training load data and the cumulative sums requiring 7, 14, 21, and 28 d respectively, the overall *n* for each variable is varied.

### Time-Loss Incidence Definitions

All time-loss incidences sustained were categorized by the club’s medical staff and were defined as any physical complaint that resulted in that individual being unable to take full part in any field- or gym-based training session or match, in line with the consensus statement defined by the International Rugby Board in 2007 (Fuller et al., 2008). Further information on the nature of the time-loss incidence was recorded, including severity (number of days unavailable for training and/or matches), the nature of the injury (contact, non-contact or illness) and the session in which the injury occurred (training or match). Each time-loss incidence was entered into the database for the day on which it occurred, and subsequently was associated with the absolute match and training load, ACWR and EWMA for that day.

### Statistical Analysis

The first section of the results presents descriptive data. To assess the impact of each match and training load quantification method on time loss-incidence occurrence, mixed effect models were conducted using the *glmer* function in R^1^ (as suggested by Windt et al., 2018). All models were fit with a Bernoulli outcome distribution (i.e., injury or no injury) and random effects for player, season, day of the season were included in all models. To assess the effect of matches and training on time-loss incidence occurrence, this variable was included in subsequent models for that section of the results. Position (forward/back) and age were included in all models. The exponential of the parameter estimate was used to calculate the odds. Due to co-linearity between the dependent variables, it was not possible to include several variables within the same model. Thus, separate models were performed for each variable. To enable a comparison of fit between models containing different variables, all analyses were subsequently performed on a reduced dataset with an equal number of data points for all variables (*n* = 14937) and the Akaike information criterion (AIC) and Bayesian information criterion (BIC) were used to assess model fit. For all analyses statistical significance was accepted as *p* < 0.05.

## Results

A total of 474 time-loss incidences were reported across the two seasons of the study, 240 time-loss incidences were reported in season one and 234 time-loss incidences in season two. Table 1 details the total time-loss incidence, nature of the injury and the session in which the injury occurred. Across the two seasons there were a total of 31,117 exposure days, with the 474 time-loss incidences resulting in a cumulative number of 9558 days lost due to injury or illness (30.7% of total days).

**TABLE 1:** Number, nature and severity of time-loss incidences across the two seasons, expressed both as absolute numbers and a percentage of the total time-loss incidences/total injuries/contact injuries/non-contact injuries, as appropriate.

**Season**	**Total time-loss incidences**	**Contact injuries**	**Non-contact injuries**	**Illnesses**
Combined	474	237 (50.0%)	165 (34.8%)	72 (15.2%)
Season one	240	125 (52.1%)	76 (31.7%)	39 (16.2%)
Season two	234	112 (47.9%)	89 (38.0%)	33 (14.1%)

**Season**	**Total injuries**	**Match injuries**	**Training injuries**	

Combined	402	257 (63.9%)	145 (36.1%)	
Season one	201	132 (65.7%)	69 (34.3%)	
Season two	201	125 (62.2%)	76 (37.8%)	

**Season**	**Contact injuries**	**Contact injuries in matches**	**Contact injuries in training**	

Combined	237	205 (86.5%)	32 (13.5%)	
Season one	125	106 (84.8%)	19 (15.2%)	
Season two	112	99 (88.4%)	13 (11.6%)	

**Season**	**Non-contact injuries**	**Non-contact injuries in matches**	**Non-contact injuries in training**	

Combined	165	52 (31.5%)	113 (68.5%)	
Season one	76	26 (34.2%)	50 (65.8%)	
Season two	89	26 (29.2%)	63 (70.8%)	

**Season**	**Exposure days**	**Days lost (severity)**	**Percentage days lost**	

Combined	31117	9558	30.7	
Season one	15869	4736	29.8	
Season two	15248	4822	31.6	

### Mixed Effect Models

Results of the mixed effect models that were conducted to examine the impact of each match and training load variable on time-loss incidence are presented in Table 2. In all models there was no significant main effect of age or interaction between age and the variable of interest (all *p* > 0.05), thus age was removed from all models. Furthermore, the interaction between position (forward/back) and the variables of interest were all non-significant (all *p* > 0.05), so the interactions were removed from the model. The main effects of position were however significant so were included in the analyses.

**TABLE 2:** Multilevel model characteristics.

**Variable**	**Variable effect**					**Position effect**			**Model characteristics**		
	**Intercept**	**Parameter estimate**	**Std. error**	**Odds**	***p*-value**	**Parameter estimate**	**Odds**	***p*-value**	**AIC**	**BIC**	**Number of observations**
Session RPE load^$^	−5.058	0.108	0.014	1.11	<0.001	0.280	1.32	0.043	4407	4456	23,032
ACWR session RPE load	−4.962	0.191	0.168	1.21	0.255	0.302	1.35	0.039	4042	4090	20,522
EWMA session RPE load	−5.451	0.697	0.218	2.01	0.001	0.300	1.35	0.039	4033	4081	20,522
Distance^$^	−5.380	0.013	0.002	1.01	<0.001	0.481	1.49	0.003	3341	3388	16,927
ACWR distance	−5.534	0.442	0.167	1.56	0.008	0.425	1.53	0.013	2990	3035	14,937
EWMA distance	−5.849	0.801	0.206	2.23	<0.001	0.408	1.50	0.014	2981	3026	14,937
High-speed running distance^$^	−5.131	0.190	0.058	1.21	<0.001	0.443	1.56	0.007	3364	3410	16,927
ACWR high-speed running	−5.206	0.121	0.085	1.13	0.154	0.428	1.53	0.013	2994	3040	14,937
EWMA high-speed running	−5.439	0.365	0.119	1.44	0.002	0.446	1.56	0.009	2987	3033	14,937

*Odds is the exponential of the parameter estimate and represents the time-loss incidence risk for a 1 unit increase in the variable (e.g., an increase in EWMA session RPE load from 1.0 to 2.0, etc.). Odds for position effect is the odds of time loss incidence in forwards compared to backs. ^$^The parameter estimate, standard error and odds for the absolute variables (session RPE load, distance and high-speed running distance) are presented for 100 unit increases in each variable.*

### Session RPE Load

**FIGURE 1:**
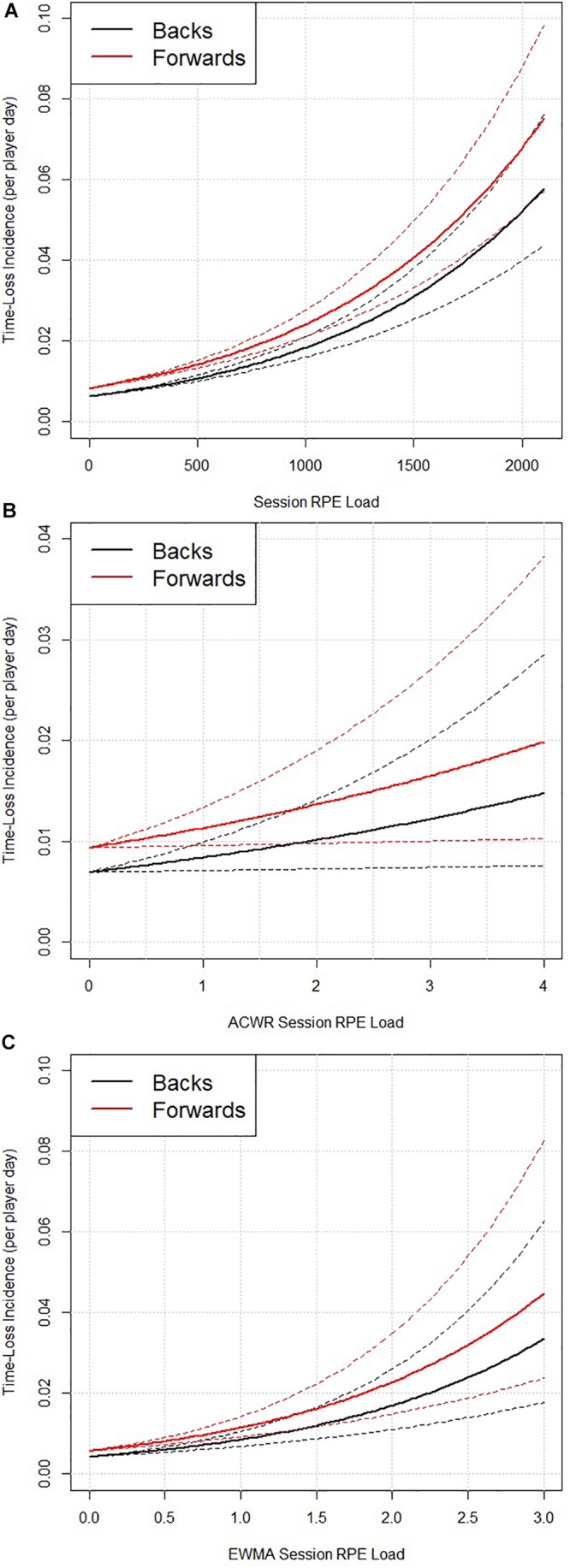
The relationship between time-loss incidence occurrence (per player day) and absolute session RPE load (*p* < 0.001) **(A)**, ACWR session RPE load (*p* = 0.255) **(B)**, and EWMA session RPE load (*p* = 0.001) **(C)**, split for backs and forwards. Data are mean ± standard error.

Session ratings of perceived exertion load demonstrated a significant influence on time-loss incidence (*p* < 0.001, Figure 1A). The odds of 1.11 indicates that for each 100 unit increase in sRPE load (e.g., from 500 to 600 AU), there was an 11% increase in time-loss incidence. The model also indicates that the odds of a time-loss incidence occurring in forwards was 1.32 compared to backs (*p* = 0.043). ACWR sRPE load did not influence time-loss incidence occurrence (*p* = 0.255, Figure 1B). However, when sRPE load was quantified using the EWMA approach, there was a significant influence on time-loss incidence (*p* = 0.001; Figure 1C). The OR of 2.01 indicates that for each 1 unit increase in EWMA sRPE load (e.g., from 0.5 to 1.5, or 1 to 2, etc.), there was a 101% increase in time-loss incidence. The model again indicates that the odds of a time-loss incidence occurring in forwards was higher (odds = 1.35) than in backs (*p* = 0.039).

### Distance

**FIGURE 2:**
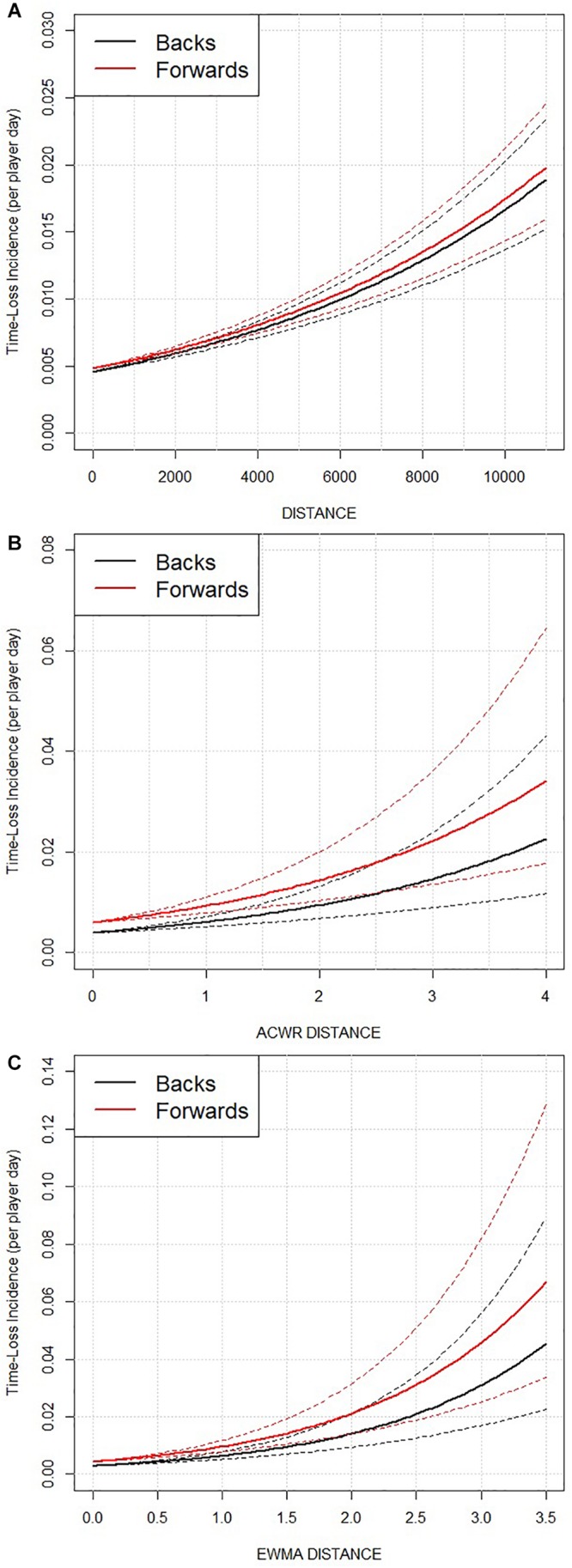
The relationship between time-loss incidence occurrence (per player day) and absolute distance (*p* < 0.001) **(A)**, ACWR distance (*p* = 0.008) **(B)**, and EWMA distance (*p* < 0.001) **(C)**, split for backs and forwards. Data are mean ± standard error.

Distance demonstrated a significant influence on time-loss incidence (*p* < 0.001, Figure 2A). The odds of 1.01 indicates that for each 100 m increase in distance covered (e.g., from 2000 to 2100 m), there was a 1% increase in time-loss incidence. The model also indicates that the odds of a time-loss incidence occurring in forwards was 1.49 compared to backs (*p* = 0.003). ACWR distance also influenced time-loss incidence (*p* = 0.008, Figure 2B), with the odds of 1.56 indicating a 56% increase in time-loss incidence with a 1 unit increase in ACWR distance (e.g., from 0.8 to 1.8). The occurrence of time-loss incidence was again greater in forwards compared to backs (odds = 1.53, *p* = 0.013). Finally, EWMA distance also demonstrated a significant influence on time-loss incidence (*p* < 0.001, Figure 2C). The OR of 2.23 indicates that for each 1 unit increase in EWMA distance (e.g., from 0.8 to 1.8), there was a 123% increase in time-loss incidence. The model again indicates that the odds of a time-loss incidence occurring in forwards was higher (odds = 1.50) than in backs (*p* = 0.014).

### High-Speed Running Distance

**FIGURE 3:**
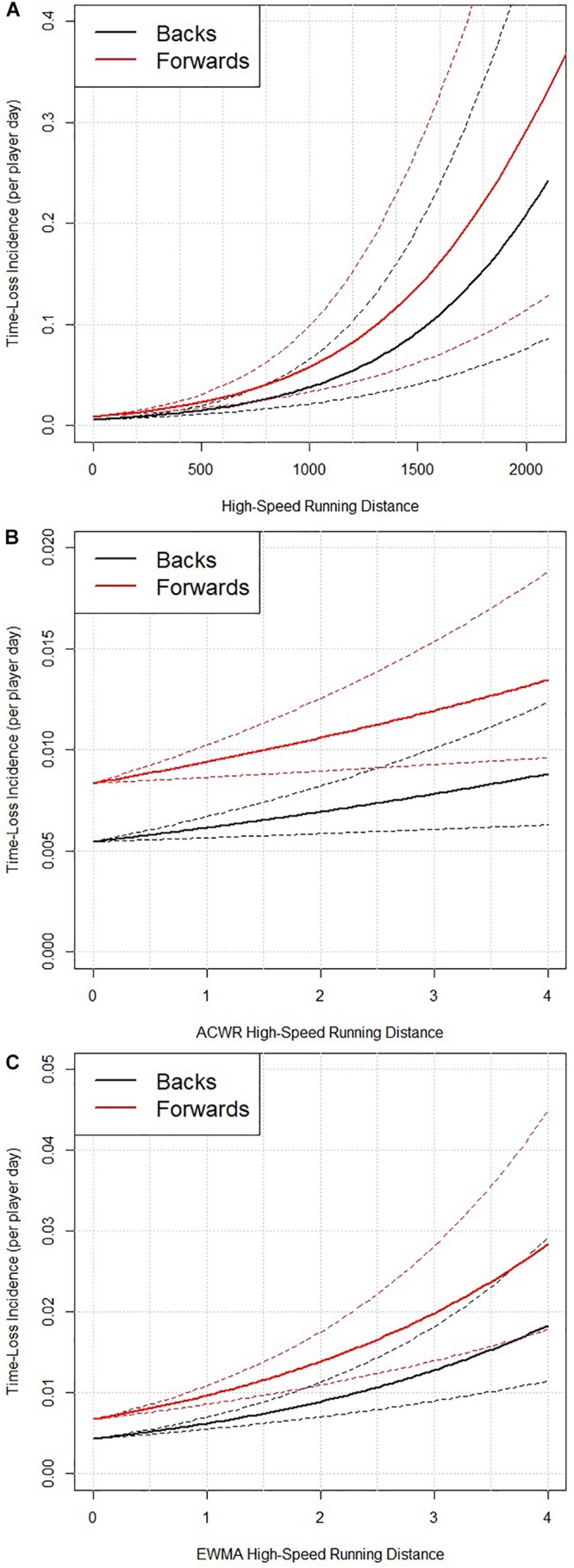
The relationship between time-loss incidence occurrence (per player day) and absolute high-speed running distance (*p* < 0.001) **(A)**, ACWR high-speed running distance (*p* = 0.154) **(B)**, and EWMA high-speed running distance (*p* = 0.002) **(C)**, split for backs and forwards. Data are mean ± standard error.

High-speed running distance also demonstrated a significant influence on time-loss incidence (*p* < 0.001, Figure 3A). The odds of 1.21 indicates that for each 100 m increase in high-speed running distance (e.g., from 800 to 900 m), there was a 21% increase in time-loss incidence. The model also indicates that the odds of a time-loss incidence occurring in forwards was 1.56 compared to backs (*p* = 0.007). However, ACWR high-speed running distance did not influence time-loss incidence (*p* = 0.154, Figure 3B). Finally, EWMA high-speed running distance demonstrated a significant influence on time-loss incidence (*p* = 0.002, Figure 3C). The OR of 1.44 indicates that for each 1 unit increase in EWMA high-speed running (e.g., from 0.8 to 1.8), there was a 44% increase in time-loss incidence. The model again indicates that the odds of a time-loss incidence occurring in forwards was higher (odds = 1.56) than in backs (*p* = 0.009).

### 7, 14, 21, and 28 d Cumulative Rolling Sums

Mixed effect models were also conducted on the 7, 14, 21, and 28 d cumulative rolling sum data for sRPE load, distance and high-speed running distance. All models returned a non-significant effect on time-loss incidence, with the exception of the 14 d cumulative rolling sum of high-speed running distance (model details: intercept = −5.3009, parameter estimate = 0.0003, standard error = 0.0001, OR (for a 1000 m increase) = 3.0, *p* = 0.040).

### Comparing Model Fit

To enable a comparison of fit between models containing different variables, all analyses were subsequently performed on a reduced dataset with an equal number of data points for all variables (*n* = 14937). This dataset was the largest possible dataset where the same number of observations for all nine variables of interest (sRPE load, distance, high-speed running distance and each of these quantified using the ACWR and EWMA approaches) were available. Models were constructed in exactly the same way as above. The AIC and BIC can be used in these models to examine which variable best explains the variance in time-loss incidence occurrence, with smaller AIC and BIC values indicative of a better model fit.

The results of this analysis are shown in Table 3. For sRPE load, distance and high-speed running distance, the absolute match and training load variables demonstrated a lower AIC and BIC than when these variables were quantified using either the ACWR or EWMA approach. This suggests that more of the variance in time-loss incidence occurrence is explained by the absolute match and training load variables rather than when the variables are quantified using either the ACWR or EWMA. Additionally, the EWMA demonstrated a lower AIC and BIC than ACWR. When comparing sRPE load, distance and high-speed running distance, the model with sRPE load had the lowest AIC and BIC, followed by distance, then high-speed running distance (Table 3).

**TABLE 3:** Multilevel model characteristics (with an equal *n* for all variables).

	**Variable effect**					**Position effect**			**Model characteristics**		
	**Intercept**	**Parameter estimate**	**Std. error**	**Odds**	***p*-value**	**Parameter estimate**	**Odds**	***p*-value**	**AIC**	**BIC**	**Number of observations**
Session RPE load^$^	−5.431	0.137	0.017	1.15	<0.001	0.407	1.50	0.019	2936	2981	14,937
ACWR session RPE load	−5.405	0.342	0.202	1.41	0.090	0.416	1.52	0.015	2993	3038	14,937
EWMA session RPE load	−6.064	1.032	0.167	2.81	<0.001	0.411	1.51	0.014	2980	3026	14,937
Distance^$^	−5.478	0.016	0.003	1.17	<0.001	0.512	1.67	0.003	2958	3004	14,937
ACWR distance	−5.534	0.442	0.167	1.56	0.008	0.425	1.53	0.013	2990	3035	14,937
EWMA distance	−5.849	0.801	0.206	2.23	<0.001	0.408	1.50	0.014	2980	3026	14,937
High-speed running distance^$^	−5.255	0.294	0.067	1.34	<0.001	0.497	1.64	0.004	2979	3025	14,937
ACWR high-speed running	−5.206	0.121	0.085	1.13	0.153	0.428	1.53	0.013	2994	3040	14,937
EWMA high-speed running	−5.439	0.365	0.119	1.44	0.002	0.446	1.56	0.009	2987	3033	14,937

*Odds is the exponential of the parameter estimate and represents the time-loss incidence risk for a 1 unit increase in the variable (e.g., an increase in EWMA session RPE load from 1.0 to 2.0, etc.). Odds for position effect is the odds of time loss incidence in forwards compared to backs. ^$^The parameter estimate, standard error and odds for the absolute variables (session RPE load, distance and high-speed running distance) are presented for 100 unit increases in each variable.*

### Calculating Time-Loss Incidence Rate

The mixed effect models provided here can be used to calculate time-loss incidence. The calculation, using sRPE load as an example, is as follows:

$$T\,i\,m\,e\,\text{-}\,l\,o\,s\,s\,i\,n\,c\,i\,d\,e\,n\,c\,e\,\left(p\,e\,r\,p\,l\,a\,y\,e\,r\,d\,a\,y\right)=\frac{\exp\left(i\,n\,t\,e\,r\,c\,e\,p\,t+p\,a\,r\,a\,m\,e\,t\,e\,r\,e\,s\,t\,i\,m\,a\,t\,e\times \text{s}\,R\,P\,E\,l\,o\,a\,d\right)}{1+\exp\left(i\,n\,t\,e\,r\,c\,e\,p\,t+p\,a\,r\,a\,m\,e\,t\,e\,r\,e\,s\,t\,i\,m\,a\,t\,e\times s\,e\,s\,s\,i\,o\,n\,R\,P\,E\,l\,o\,a\,d\right)}$$

The above calculation would be for a back. To calculate time-loss incidence in a forward the effect of position must be added to the equation, as follows:

$$T\,i\,m\,e\,\text{-}\,l\,o\,s\,s\,i\,n\,c\,i\,d\,e\,n\,c\,e\,\left(p\,e\,r\,p\,l\,a\,y\,e\,r\,d\,a\,y\right)=\frac{\begin{array}{c} \exp(\left(intercept+parameterestimate\times sRPEload\right)\\ +positionparameterestimate) \end{array}}{\begin{array}{c} 1+\exp(\left(intercept+parameterestimate\times sRPEload\right)\\ +positionparameterestimate) \end{array}}$$

For example, for a forward with a sRPE load of 650 AU, the calculation would be:

$$\frac{E\,x\,p\,\left(\left(-5.058+0.108\times 650\right)+0.280\right)}{1+e\,x\,p\,\left(\left(-5.058+0.108\times 650\right)+0.280\right)}$$

= 0.017 time-loss incidences per player day

### Time-Loss Incidence in Matches and Training

To examine the impact of matches compared to training on time-loss incidence, an additional (match or training) variable was included in the mixed effect models assessing the effect of the absolute match and training load variables on time-loss incidence. There were no interactions between the absolute match and training load variables and matches/training (sRPE load, *p* = 0.218; distance, *p* = 0.146; high-speed running distance, *p* = 0.501). However, there was a significant main effect, suggesting that time-loss incidence was greater in matches compared to training (sRPE load: parameter estimate = 2.313, standard error = 0.235, OR = 10.1, *p* < 0.001; distance: parameter estimate = 2.479, standard error = 0.241, OR = 11.9, *p* < 0.001; high-speed running distance: parameter estimate = 2.732, standard error = 0.001, OR = 15.4, *p* < 0.001).

## Discussion

The aim of the present study was to identify the best predictor of time-loss incidence occurrence between absolute match and training load variables, the ACWR and the EWMA quantification methods, when applied to sRPE load, distance and high-speed running distance. The main findings of the present study suggest that changes in the absolute match and training load variables (sRPE load, distance and high-speed running distance), with no quantification method applied to them, provide the best method of explaining the variance in time-loss incidence rate in elite Rugby Union players. Specifically, the use of absolute sRPE load provided the lowest AIC and BIC values, followed by distance and then high-speed running distance. As shown in Tables 2, 3, when comparing the different match and training load quantification methods, the EWMA method better explained the variance in time-loss incidence occurrence than the ACWR method, as the AIC and BIC were lower across all variables for EWMA compared to ACWR. A higher time-loss incidence was seen in forwards compared to backs, ranging from 32% (sRPE load) to 62% (distance), but no interaction was seen between position and any match and training load variables. The models examining cumulative rolling sums did not identify any significant effects on time-loss incidence rate of these variables, with the exception being 14 d cumulative rolling sum for high-speed running distance. Overall, these findings suggest that the absolute match and training load variables may provide the best predictors of time-loss incidence rates, with sRPE load likely to be the optimal variant of those examined here.

This is the first study to compare absolute match and training load, ACWR and EWMA methods for the assessment of time-loss incidence in elite athletes. The model fit assessment suggests that the absolute match and training load variables (sRPE load, distance and high-speed running distance) are better predictors of time-loss incidence occurrence in professional Rugby Union players, compared to when the same variables are quantified using the ACWR and EWMA approaches. Furthermore, it appears that sRPE load was the best variable to use to assess time-loss incidence (when compared to distance and high-speed running distance). Unlike the GPS-derived variables (distance and high-speed running distance) which require expensive technologies to collect, sRPE load provides performance and medical staff with a low cost, easy to administer method of match and training load assessment and management (Kraft et al., 2014). It is also possible that the RPE variable provides a more accurate reflection of contacts and collisions during Rugby Union (not picked up by GPS variables). Furthermore, RPE was also recorded during gym-based sessions where GPS monitoring is not possible, a further potential explanation of the enhanced predictive ability of the models with RPE included. Additionally, the calculations provided within this paper provide performance and medical staff with actionable values which can be easily communicated to coaches when assessing an individual players risk; and thus, enable them to make an informed decision about player match and training load.

When the two ratio quantification methods (ACWR and EWMA) are compared, the EWMA approach better explains the variance in time-loss incidence occurrence compared to the ACWR method, as shown through the lower AIC and BIC values. This therefore affirms the thoughts of Menaspà (2017) and Williams et al. (2017) who suggest the ACWR approach lacks sensitivity and suffers mathematical coupling (Lolli et al., 2017). Furthermore, it agrees with the findings of Murray et al. (2017), who investigated the relationship between match and training load and injury in Australian footballers using only objective (GPS) measures and quantified it using both the ACWR and EWMA. The present study extends these findings to both subjective (sRPE load) and objective measures and utilized an individual approach to determining high-speed running distance (>70% of an individual’s maximum velocity), compared to the set parameters (18–24 km/h) used by Murray et al. (2017). When assessing the cumulative rolling sums models, the only variable to return a significant effect on time-loss incidence rate was 14 d high-speed running distance, therefore, aggregating match and training load into weekly sums does not further assist in time-loss incidence occurrence assessment. To summarize, the absolute match and training load variables better explain the variance in time-loss incidence rates above the ratio ACWR and EWMA methods and the cumulative rolling sums approach.

The time-loss incidence curves describing the relationships between time-loss incidence and the match and training load variables are shown in Figures 1–3. In contrast to previous work by Blanch and Gabbett (2015), who suggested that a U-shaped pattern existed between injury incidence and an ACWR. Our analysis and models do not find any evidence of this form of U-shape pattern. This apparent disagreement in findings may have arisen because the independent variables in the Blanch and Gabbett (2015) analysis appears to be based on aggregated categorical data from a series of research investigations (Hulin et al., 2014, 2016) whereas in the current study the models use the raw/absolute match and training load data from each player on each day.

Another novel aspect of this study was the comparison in time-loss incidence rates between forwards and backs. Players occupying forward positions were found to have a higher time-loss incidence rates compared to backs for all match and training load variables, ranging from 32 to 62%. An explanation for this may be due to the higher involvement of total impacts, tackles and rucks of forwards compared to backs as shown by Lindsay et al. (2015), and also demonstrated in the results of this paper the number of time-loss incidence occurring through contact injuries makes up 50.0% of all time-loss incidences. Furthermore, it is important to note that position did not interact with any of the match and training load variables, thus suggesting that time-loss incidence rates changed with increased match and training load in a similar manner for both positional groups. In addition, the present study also examined time-loss incidence in training compared to matches. The findings suggest that the likelihood of a time-loss incidence occurring was 10–15 times higher in matches compared to training. However, none of the absolute match and training load variables interacted with the training/match variable, suggesting that the increased time-loss incidence was similar when load increased in both training and matches by a similar amount.

### Practical Applications in Rugby Union

The “sweet-spot” of an ACWR of 0.8–1.3 (based on sRPE load data) suggested by Blanch and Gabbett (2015) has been widely cited and used within professional sport. However, the findings of the present study suggest that the absolute match and training load variables provide a better explanation of the variance in time-loss incidence (and thus should be incorporated in load management models to minimize the time-loss incidences occurring), when compared to the more commonly used ACWR and EWMA approaches. The present study enhances previous work in the area (Rogalski et al., 2013; Cross et al., 2016; Hulin et al., 2016) by showing that subjective measures (i.e., sRPE load) can be quantified in various ways to manage time-loss incidence. Session RPE load is a relatively inexpensive method when compared to the GPS-derived variables. However, there are obvious challenges associated with the collection of sRPE load data for every player for every session, particularly within 30 min of the end of each session. It should be noted however that evidence has suggested that sRPE is still valid up to 24 h post-exercise (Phibbs et al., 2017), potentially further enhancing the practical utility of sRPE as a monitoring tool. The additional inclusion of objective GPS-based measures can add further value to sRPE load alone by assisting the load management processes due to its capabilities of providing live feedback during training sessions for at risk individuals (e.g., those returning from injury), and may be easier to collect in a large number of players at one time.

### Limitations and Future Research

The findings of the present study are based on data from one professional Rugby Union club thus the applicability to all clubs is unknown. Future work could build upon this by, for example, including match and training load and time-loss incidence data from multiple clubs. Furthermore, future work could also consider the relationship between match and training load and different types of time-loss incidence (i.e., contact injuries, non-contact injuries and illness) and whether the injury occurred in training or matches separately. This could potentially allow for greater resolution between variables and quantification methods. Future work, perhaps with multiple clubs over multiple seasons, could also consider matches in isolation, to examine whether any aspects of matches (e.g., time of day, match outcome, etc.) influence time-loss incidence risk. However, achieving this volume of data from multiple clubs, allowing such analysis to be undertaken, will be challenging, not least due to the variations in the measurement and management of match and training load and time-loss incidence between clubs. A further potential limitation of the current study was the use of different GPS monitoring systems from season one to season two, as stated in the materials and methods section. Future work should endeavor to use the same GPS monitoring system for the duration of the data collection process to avoid potential conflicts between units.

## Conclusion

The match and training load variable that best explains the variance in time-loss incidence was absolute sRPE load, followed by absolute distance and absolute high-speed running distance. These findings therefore suggest that the use of absolute match and training load data from each player on each day may be more beneficial when assessing time-loss incidence risk, when compared to the more commonly used ACWR and EWMA quantification approaches. The objective GPS-derived variables still appeared to provide a significant explanation of the variance in time-loss incidence occurrence, and thus the use of GPS as a real-time monitoring tool (providing live feedback) means that such measures may well have applied utility. When assessing the quantified match and training load variables (ACWR and EWMA), the EWMA variables better explained the variance in time-loss incidence compared to the ACWR method. No relationship was seen between the 7, 14, 21, and 28 d cumulative rolling sums for all variables (sRPE load, distance and high-speed running), with the exception of 14 d cumulative rolling sum of high-speed running distance. Finally, the time-loss incidence curves derived from the mixed effect models (for all absolute, ACWR and EWMA variables) did not show a U-shaped pattern. Overall, these findings suggest that the absolute match and training load variables provide the best predictors of time-loss incidence rates, with sRPE load likely to be the optimal variant of those examined here. Furthermore, the EWMA approach to quantifying match and training load was a better predictor of time-loss incidence risk than when the same variables were calculated using the ACWR approach.

## Ethics Statement

This study was carried out in accordance with the recommendations of the non-invasive ethics committee of the School of Science and Technology at Nottingham Trent University with written informed consent from all participants. All subjects gave written informed consent in accordance with the Declaration of Helsinki. The protocol was approved by the non-invasive ethics committee of the School of Science and Technology at Nottingham Trent University.

## Author Contributions

BC collected and analyzed the data, and drafted the manuscript. SC and JM assisted with data analysis and drafting the manuscript. CS and AB contributed to drafting the manuscript. GS provided statistical expertise and advice on the data analysis. All authors have read and approved the final manuscript.

## Conflict of Interest

The authors declare that the research was conducted in the absence of any commercial or financial relationships that could be construed as a potential conflict of interest.

## Footnotes

1 www.r-project.org

## References

[B1] Adams J. Kirkby R. (2001). Exercise dependence and overtraining: the physiological and psychological consequences of excessive exercise. *Sports Med. Train. Rehab.* 10 199–222. 10.1080/10578310210395

[B2] Beato M. Coratella G. Stiff A. Iacono A. D. (2018). The validity and between-unit variability of GNSS units (STATSports Apex 10 and 18 Hz) for measuring distance and peak speed in team sports. *Front. Physiol.* 9:1288. 10.3389/fphys.2018.01288 30298015 PMC6161633

[B3] Blanch P. Gabbett T. J. (2015). Has an athlete trained enough to return to play safely? the acute:chronic workload ratio permits clinicians to quantify a player’s risk of subsequent injury. *Br. J. Sports Med.* 50 471–475. 10.1136/bjsports-2015-095445 26701923

[B4] Bowen L. Gross A. S. Gimpel M. Li F.-X. (2016). Accumulated workloads and the acute: chronic workload ration relate to injury risk in elite youth football players. *Br. J. Sports Med.* 51 452–459. 10.1136/bjsports-2015-095820 27450360 PMC5460663

[B5] Carling C. Le Gall F. McCall A. Nédélec M. Dupont G. (2015). Squad management, injury and match performance in a professional soccer team over a championship- winning season. *Eur. J. Sport Sci.* 15 573–582. 10.1080/17461391.2014.955885 25216043

[B6] Colby M. J. Dawson B. Heasman J. Rogalski R. Gabbett T. J. (2014). Accelerometer and GPS-derived running loads and injury risk in elite australian footballers. *J. Strength Cond. Res.* 28 2244–2252. 10.1519/JSC.0000000000000362 25054573

[B7] Cross M. J. Williams S. Trewartha G. Kemp S. P. Stokes K. A. (2016). The influence of in-season training loads on injury risk in professional rugby union. *Int. J. Sports Physiol. Perform.* 11 350–355. 10.1123/ijspp.2015-2187 26309331

[B8] Drew M. K. Purdam C. (2016). Time to bin the term ‘overuse’ injury: is ‘training load error’ a more accurate term? *Br. J. Sports Med.* 50 1423–1424. 10.1136/bjsports-2015-095543 26843537

[B9] Foster C. Florhaug J. A. Franklin J. Gottschall L. Hrovatin L. A. Parker S. (2001). A new approach to monitoring exercise training. *J. Strength Cond. Res.* 15 109–115. 11708692

[B10] Fuller C. W. Laborde F. Leather R. J. Molloy M. G. (2008). International rugby board rugby world cup 2007 injury surveillance study. *Br. J. Sports Med.* 42 452–459. 10.1136/bjsm.2008.047035 18539659

[B11] Gabbett T. (2004). Influence of training and match intensity on injuries in rugby league. *J. Sports Sci.* 22 409–417. 10.1080/02640410310001641638 15160594

[B12] Gabbett T. J. Domrow N. (2007). Relationships between training load, injury, and fitness in sub-elite collision sport athletes. *J. Sports Sci.* 25 1507–1519. 10.1080/02640410701215066 17852696

[B13] Gabbett T. J. Ullah S. (2012). Relationships between running and soft-tissue injury in elite team sport athletes. *J. Strength Cond. Res.* 26 953–960. 10.1519/JSC.0b013e3182302023 22323001

[B14] Hulin B. T. Gabbett T. J. Blanch P. Chapman P. Bailey D. Orchard J. W. (2014). Spikes in acute workload are associated with increased injury risk in elite cricket fast bowlers. *Br. J. Sports Med.* 48 708–712. 10.1136/bjsports-2013-092524 23962877

[B15] Hulin B. T. Gabbett T. J. Lawson D. W. Caputi P. Sampson J. A. (2016). The acute:chronic workload ratio predicts injury: high chronic workload may decrease injury risk in elite rugby league players. *Br. J. Sports Med.* 50 231–236. 10.1136/bjsports-2015-094817 26511006

[B16] Impellizzeri F. M. Rampinini E. Coutts A. J. Sassi A. Marcora S. M. (2004). Use of RPE-based training load in soccer. *Med. Sci. Sports Exerc.* 36 1042–1047. 10.1249/01.mss.0000128199.23901.2f 15179175

[B17] Kraft J. A. Green J. M. Thompson K. R. (2014). Session ratings of perceived exertion responses during resistance training bouts equated for total work but differing in work rate. *J. Strength Cond. Res.* 28 540–545. 10.1519/JSC.0b013e31829b569c 24476745

[B18] Lindsay A. Draper N. Lewis J. Gieseg S. P. Gill N. (2015). Positional demands of professional rugby. *Eur. J. Sports Sci.* 15 480–487. 10.1080/17461391.2015.1025858 25830235

[B19] Lolli L. Batterham A. M. Hawkins R. Kelly D. M. Strudwick A. J. Thorpe R. (2017). Mathematical coupling causes spurious correlation within the conventional acute-to-chronic workload ratio calculations. *Br. J. Sports Med.* 53 921–922. 10.1136/bjsports-2017-098110 29101104

[B20] Lolli L. Batterham A. M. Hawkins R. Kelly D. M. Strudwick A. J. Thorpe R. (2018). The acute-to-chronic workload ratio: an inaccurate scaling index for an unnecessary normalisation process? *Br. J. Sports* 13:bjsorts-2017-098884. 10.1136/bjsports-2017-098884 29899049

[B21] Menaspà P. (2017). Are rolling averages a good way to assess training load for injury prevention? *Br. J. Sports. Med.* 51 618–619. 10.1136/bjsports-2016-096131 27222309

[B22] Murray N. B. Gabbett T. J. Townshend A. D. Blanch P. (2017). Calculating acute:chronic workload ratios using exponentially weighted moving averages provides a more sensitive indicator of injury likelihood than rolling averages. *Br. J. Sports Med.* 51 749–754. 10.1136/bjsports-2016-097152 28003238

[B23] Phibbs P. J. Roe G. Jones B. Read D. B. Weakley J. Darrall-Jones J. (2017). Validity of daily and weekly self-reported training load measures in adolescent athletes. *J. Strength Cond. Res.* 31 1121–1126. 10.1519/JSC.0000000000001708 28328718

[B24] Rogalski B. Dawson B. Heasman J. Gabbett T. J. (2013). Training and game loads and injury risk in elite Australian footballers. *J. Sci. Med. Sport* 16 499–503. 10.1016/j.jsams.2012.12.004 23333045

[B25] Thornton H. R. Nelson A. R. Delaney J. A. Serpiello F. R. Duthie G. M. (2019). Interunit reliability and effect of data-processing methods of global positioning systems. *Int. J. Sports Physiol. Perform.* 14 432–438. 10.1123/ijspp.2018-2273 30204529

[B26] Waldron M. Worsfold P. Twist C. Lamb K. (2011). Concurrent validity and test-retest reliability of a global positioning system (GPS) and timing gates to assess sprint performance variables. *J. Sports. Sci.* 29 1613–1619. 10.1080/02640414.2011.608703 22004326

[B27] Williams S. Trewartha G. Kemp S. Stokes K. (2013). A meta-analysis of injuries in senior men’s professional rugby union. *Sports Med.* 43 1043–1055. 10.1007/s40279-013-0078-1 23839770

[B28] Williams S. Trewartha G. Kemp S. P. T. Brooks J. H. M. Fuller C. W. Taylor A. E. (2015). Time loss injuries compromise team success in elite rugby union: a 7-year prospective study. *Br. J. Sports Med.* 50 651–656. 10.1136/bjsports-2015-094798 26552415

[B29] Williams S. West S. Cross M. J. Stokes K. A. (2017). Better way to determine the acute:chronic workload ratio? *Br. J. Sports Med.* 51 209–210. 10.1136/bjsports-2016-096589 27650255

[B30] Windt J. Ardern C. L. Gabbett T. J. Khan K. M. Cook C. E. Sporer B. C. (2018). Getting the most out of intensive longitudinal data: a methodological review of workload-injury studies. *BMJ Open* 8:e022626. 10.1136/bmjopen-2018-022626 30282683 PMC6169745

